# Overseas immigration of fall armyworm, *Spodoptera frugiperda* (Lepidoptera: Noctuidae), invading Korea and Japan in 2019

**DOI:** 10.1111/1744-7917.12940

**Published:** 2021-10-15

**Authors:** Ming‐Fei Wu, Guo‐Jun Qi, Hui Chen, Jian Ma, Jie Liu, Yu‐Ying Jiang, Gwan‐Seok Lee, Akira Otuka, Gao Hu

**Affiliations:** ^1^ College of Plant Protection Nanjing Agricultural University Nanjing 210095 China; ^2^ Guangdong Provincial Key Laboratory of High Technology for Plant Protection/Plant Protection Research Institute Guangdong Academy of Agricultural Sciences Guangzhou 510640 China; ^3^ Division of Pest Forecasting China National Agro‐Tech Extension and Service Center Beijing 100026 China; ^4^ Department of Agro‐food Safety and Crop Protection National Institute of Agricultural Sciences Wanju 55365 Korea; ^5^ Institute of Agricultural Machinery National Agriculture and Food Research Organization Tsukuba 3058517 Japan

**Keywords:** Asian migration arena, atmospheric circulation, *Spodoptera frugiperda*, trajectory analysis windborne insect migration

## Abstract

The fall armyworm (FAW), *Spodoptera frugiperda* (J.E. Smith), spread rapidly in Africa and Asia recently, causing huge economic losses in crop production. Fall armyworm caterpillars were first detected in South Korea and Japan in June 2019. Here, the migration timing and path for FAW into the countries were estimated by a trajectory simulation approach implementing the insect's flight behavior. The result showed that FAWs found in both South Korea and Japan were estimated to have come from eastern China by crossing the Yellow Sea or the East China Sea in 10–36 h in three series of migrations. In the first series, FAW moths that arrived on Jeju Island during 22–24 May were estimated to be from Zhejiang, Anhui and Fujian Provinces after 1–2 nights’ flights. In the second series, it was estimated that FAW moths landed in southern Korea and Kyushu region of Japan simultaneously or successively during 5–9 June, and these moths mostly came from Guangdong and Fujian Provinces. The FAW moths in the third series were estimated to have immigrated from Taiwan Province onto Okinawa Islands during 19–24 June. During these migrations, southwesterly low‐level jets extending from eastern China to southern Korea and/or Japan were observed in the northwestern periphery of the western Pacific Subtropical High. These results, for the first time, suggested that the overseas FAW immigrants invading Korea and Japan came from eastern and southern China. This study is helpful for future monitoring, early warning and the source control of this pest in the two countries.

## Graphical abstract

Fall armyworm (FAW) would take a round‐trip migratory cycle in eastern Asia, where they will pose a serious threat to food security. Therefore, it is very important to understand the migration path and the source area for monitoring and controlling this pest. This study, for the first time, suggested that the overseas FAW immigrants invading Korea and Japan came from eastern and southern China. This study is helpful for future monitoring, early warning and the source control of this pest in the two countries.

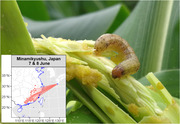

## Introduction

The fall armyworm (FAW), *Spodoptera frugiperda* (J.E. Smith), native to the tropical and subtropical regions of the Americas, is a serious invasive agricultural pest (Luginbill, 1928; Sparks, 1979). With its strong long‐distance flight capability, fecundity and adaptability, FAW can spread rapidly and cause serious damage to crop production in suitable environments (Johnson, 1987; Early *et al*., 2018; Rwomushana *et al*., 2018; Yang *et al*., 2021). Its caterpillars can feed on over 350 plant species, such as corn, sorghum, millet and barley (Montezano *et al*., 2018), causing huge economic losses in crop production, particularly that of maize. Typically, yield losses of between 15%–73% are caused by FAW outbreaks on corn (Murúa *et al*., 2006; Lima *et al*., 2010; Rodríguez‐del‐Bosque *et al*., 2012; Ayala *et al*., 2013).

The fall armyworm has long been a major agricultural problem in the Western Hemisphere until 2016 (Stokstad *et al*., 2017; Rwomushana *et al*., 2018). With the rapid expansion of global trade, FAW first invaded Nigeria in 2016 (Goergen *et al*., 2016; Cock *et al*., 2017), its first major introgression into the Eastern Hemisphere (Nagoshi *et al*., 2018). It spread rapidly across Africa and destroyed maize fields with remarkable speed (Stokstad, 2017). In May 2018, FAW was reported to be detected in India (Sharanabasappa *et al*., 2018), and then it was widely found in all countries in South, Southeast and East Asia in the next 2 years (Jiang *et al*., 2019; Ma *et al*., 2019). It was reported that FAW's first invasion of Korea occurred on Jeju Island in June 2019 (Lee *et al*., 2020), and the Plant Protection Station of Japan announced on 3 July 2019 that larvae found in Kagoshima in late June were confirmed to be FAWs (Ministry of Agriculture, Forestry and Fisheries, 2019), causing a potential threat to its corn production and food security. Even worse, invasive FAW has developed resistance to several synthetic insecticides (Guan *et al*., 2021).

With the aid of high‐altitude winds, FAW can fly hundreds of kilometers over several nights (Rose *et al*., 1975; Westbrook *et al*., 2016). Long‐distance migration is the means by which FAW has rapidly expanded throughout its new invasive regions and produced large‐scale outbreaks. In fact, eastern Asia is a very suitable region for insect migration, and there are many migratory insects, some of which are serious crop pests, including rice planthoppers (*Nilaparvata lugens* and *Sogatella furcifera*) and the oriental armyworm (*Mythimna separata*) (Feng *et al*., 2008; Hu *et al*., 2019, 2017). When FAW was first detected in Yunnan Province, China, its migration paths in eastern Asia were soon predicted by a trajectory analytical approach in several previous studies (Ma *et al*., 2019; Wu *et al*., 2019; Chen *et al*., 2020; Li *et al*., 2020). FAW cannot survive in winter in most areas of China, Japan and Korean Peninsula, but it was highly likely that Asian populations would evolve annual spring northward migrations into these above regions (and presumably south again the following autumn) (Chen *et al*., 2020; Li *et al*., 2020). For instance, FAW was predicted to migrate into Japan and Korea from East China during monsoon season between 1 June and 15 July 2019 (Ma *et al*., 2019). Conformation of these predictions was provided by the fact that the simulated migration paths and migratory ranges in these previous studies were in accordance with its spread trends in China, Korea and Japan in 2019 (Jiang *et al*., 2019; Ma *et al*, 2019; Ministry of Agriculture, Forestry and Fisheries, 2019; Lee *et al*., 2020; Li *et al*., 2020).

Migration and colonization between southeastern Asia and eastern Asia would result in the emergence of a round‐trip migratory cycle of FAW. The Japanese Islands and the Korean Peninsula face a significant threat to their food security and agricultural productivity from the invasion of FAW. Hence, a better understanding of migration paths and the source areas of FAW immigrants will be beneficial to monitoring, early warning and the source controlling of this pest in Japan and Korea. In this study, for the first time, migratory paths and weather backgrounds for overseas migrations of FAW invading Korea and Japan were simulated using a numerical weather simulation model and a trajectory analysis program (Wang *et al*., 2017; Wu *et al*., 2018; Li *et al*., 2020) with the moth's flight behavior implemented. This study presents a scientific basis for guiding monitoring and management work to control FAW in Korean peninsula and Japan.

## Materials and methods

### Larva occurrence survey

**Fig. 1:**
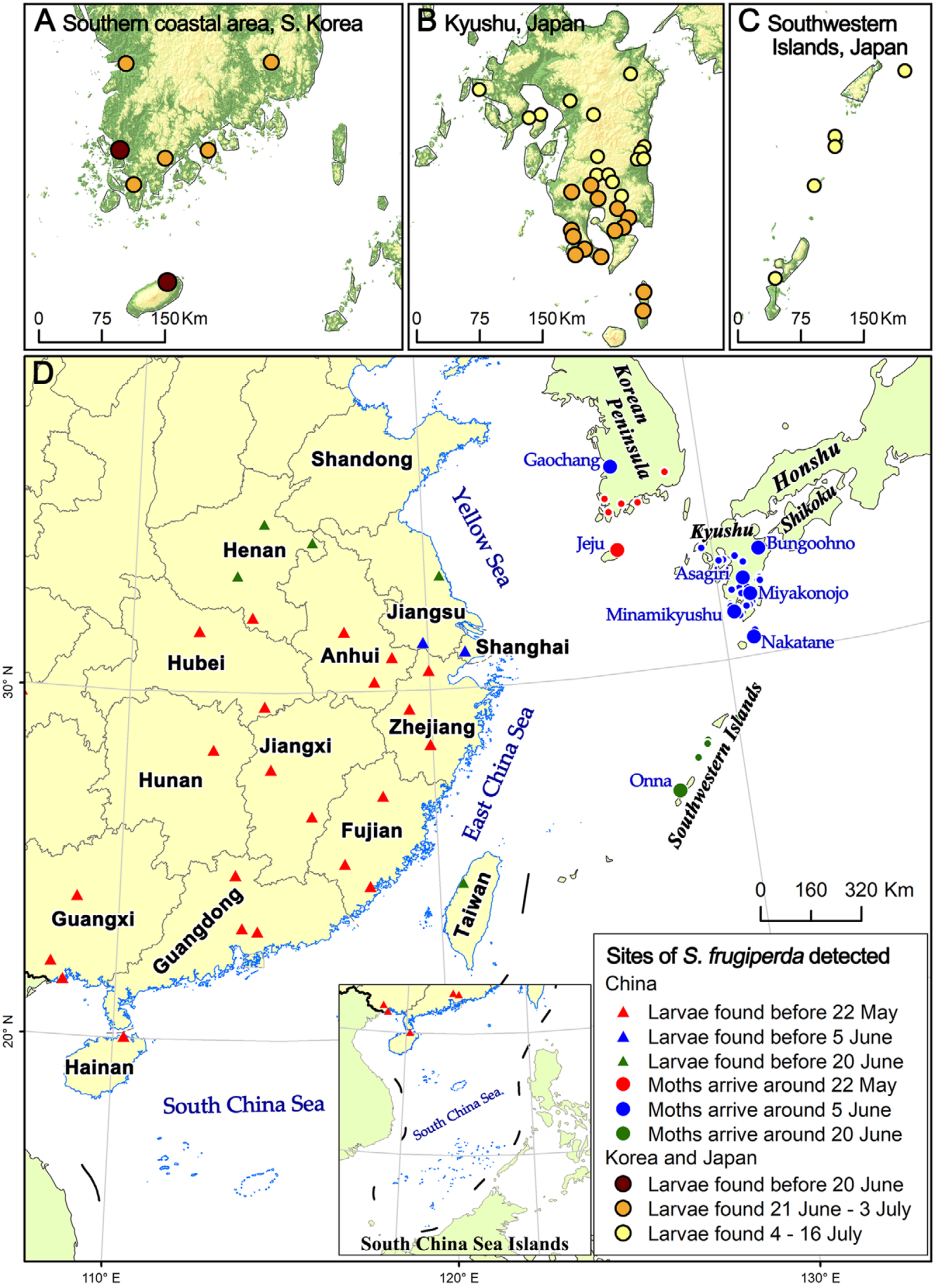
Map of the study area in East Asia. The Korean Peninsula and islands of Japan as well as east China were parts of many animals’ migration pathways in eastern Asia. Caterpillars of *S. frugiperda* were found in southern Korea and southwestern Japan in June and July 2019 (A, B and C). Their sites are shown in circled points; among these sites, bigger solid circles in (D) have been analyzed in detail. These caterpillars were also distributed in southern and eastern China, such as Anhui, Fujian, Zhejiang, Jiangxi, Guangdong and Taiwan Provinces. Chinese sites of *S. frugiperda*’s larvae found are shown in solid triangles (D). The possible arrival date of FAW moths at each site in Korea and Japan, and the emergence date of FAW moths at each site in China were estimated based on day‐degree model. GS(2021)3135.

In Korea, field surveys to find FAW larvae were conducted by national and provincial organizations since early June (Fig. 1). The first unfamiliar larval samples on young sweet corn were collected by experts of the Jeju Agricultural Extension and Service on Jeju Island, southern Korea in early June, and identified as FAWs based on morphological and genetic information by the National Institute of Agricultural Sciences (Lee *et al*., 2020). Then, an intensive field survey to find larvae mostly on sweet corn, first on the island, then nationwide, was conducted. In Japan, field surveys to find larvae were conducted by prefectural plant protection stations since late June 2019 (Fig. 1). All possible hosts, including maize, rice, sweet potato, sorghum, sugarcane and Poaceae grass, were surveyed. Larvae samples were collected and identified based on their morphology and/or genetic information by each regional station of the Plant Protection Station of Japan. Larval stage was estimated by the size of the body and head capsule of the collected larvae. To analyze first and early immigrations only, larval survey data until mid‐July in two countries were used.

The survey data of FAW in China were obtained from the National Agro‐Tech Extension and Service Center, and this data include date of collection, host plant and the age of FAW first detected in each county. China survey data were used to verify FAW possible source areas that was estimated by trajectory approach.

### Estimation of FAW arrival date based on degree‐day model

There is no report on FAW's ability of diapausing, and its generation stage is closely related to local ambient temperatures. Based on Schlemmer's study (2018), the minimum temperature thresholds (°C) for the development of FAW egg, 1st–6th instar larvae and pupa were 13.01, 8.49, 12.17, 13.47, 13.11, 13.13, 14.85 and 13.24, respectively, and their cumulative degree‐days for each development stage were 35.73, 47.14, 26.86, 21.58, 24.78, 28.53, 32.57 and 147.06, respectively. Development periods from egg to larval ages at the time of being found in the field were estimated by calculating degree‐day values. Then, possible arrival dates at each site were estimated based on the instar of the oldest larva; the latest possible arrival date was calculated by assuming the oldest larva just molt from its previous instar while the earliest date by assuming the oldest larva would molt into next instar soon. Here, degree‐day values were calculated with the average of the daily 2‐m air temperature data from the FNL datasets (https://psl.noaa.gov/data/reanalysis/reanalysis.shtml). At Japanese sites, only old caterpillars were found in fields, but no young ones were seen. Therefore, we suspected that some insects had already turned into the pupa stage and hid in the soil. Since an adult can survive on average to approximately 10 d and even up to 21 d (Luginbill, 1928), a possible arrival date was able to be moved by 10 d or more earlier. With the same degree‐day method, possible emerging and emigrating dates in eastern China were estimated.

### Weather simulation and meteorological data processing

The Weather Research and Forecasting Model is an advanced meso‐scale numerical weather prediction system, which provided hourly high‐resolution meteorological background for the trajectory analysis (Skamarock *et al*., 2019). Final Analysis (FNL) data from the National Centers for Environmental Prediction (NCEP) and National Center for Atmospheric Research (NCAR) were used as meteorological data for model input. FNL data is a 6‐hourly, global, 1‐degree grid meteorological dataset. In this study, the horizontal dimension of a model domain was 140 × 150 grid points at a resolution of 30 km. Twenty‐nine vertical layers were available and the model ceiling was set to be 100 hPa. The model forecast time was 72 h, and simulated meteorological data of horizontal and vertical wind speeds, temperature and precipitation were output at intervals of 1 h. The calculation schemes and model parameters were the same as our previous study (Ma *et al*., 2019).

### Trajectory modeling of FAW

The trajectory analysis method has been widely applied in simulating the flight trajectory of many seasonal migratory pests such as *Cnaphalocrocis medinalis*, *M. separata*, *N. lugens*, *Helicoverpa zea* (Otuka, 2005; Hu *et al*., 2013, 2014; Wang *et al*., 2017; Ma *et al*., 2018; Wu *et al*., 2018) and also in FAW (Ma *et al*., 2019; Li *et al*., 2020; Luo *et al*., 2020; Wu *et al*., 2021). The parameters of this study are consistent with previous studies (Ma *et al*., 2019; Chen *et al*., 2020; Li *et al*., 2020), and main flight characteristics and parameters are as follows:

- Noctuid moths fly at night, typically take off at dusk and terminate their night flights at the following dawn (Chapman *et al*., 2012, 2010; Wang *et al*., 2017). According to the sunrise and sunset times at departure and landing points in summer, FAW can fly continuously for 10 h every night and mostly fly for 3 consecutive nights, similar to most other noctuid moths whenever flying over land (Wang *et al*., 2017).
- We did not explore moths’ vertical movements during flight or takeoff and landing processes. Instead, to ensure we would capture most probable flight heights, we assumed eight possible constant flight heights: 500, 750, 1000, 1250, 1500, 1750, 2000 and 2250 m above mean sea level (Tojo *et al*., 2013; Wang *et al*., 2017; Ma *et al*., 2019; Li *et al*., 2020).
- Other similar‐sized noctuid moths fly at a self‐powered flight speed of approximately 2.5–4 m/s (Drake & Reynolds, 2012; Minter *et al*., 2018). Thus, we assumed a flight speed of 3.0 m/s in our model (Li *et al*., 2020). As we do not know if the Asian FAW moths have a preferred flight heading, we assumed that the flight vector will be aligned with the downwind direction (Li *et al*., 2020).
- Our preliminary flight ability test found that FAW stopped flying after a short period when the ambient temperature was around 13.8 °C (H. Chen, Nanjing Agricultural University, unpublished data). Therefore, it was assumed that FAW cannot fly when the ambient air temperature at its flight altitude falls below 13.8 °C, the minimum temperature for flight (Hogg *et al*., 1982; Ma *et al*., 2019; Li *et al*., 2020). Therefore, if it gets lower than the threshold, then the trajectory was terminated.

Start points of backward trajectories with different starting heights were set over locations where FAW larvae were found in South Korea and Japan. When moths migrated over the Yellow Sea and East China Sea towards South Korea and Japan, continuous flight duration was allowed to exceed 10 h due to a longer distance. Thus, we could not know specific landing times. Therefore, backward trajectories were calculated hourly on arriving dates estimated above, and terminated when: (i) flight duration became 36 h; (ii) air temperature at the flight height dropped below 13.8 °C; or (iii) reached over the land (possible source) at dusk (19:00 h Korea/Japan Standard Time, same hereinafter). Trajectories that reached over the land at dusk were considered as valid. Otherwise, trajectories that were terminated due to the duration and temperature limits were considered as invalid. The moths were assumed to take a set of multiple migrations in up to 3 consecutive nights, valid end‐points of the previous backward trajectories were set as new starting points to calculate the following backward trajectories; these new trajectories were started at sunrise (5:00 h) and terminated at dusk (19:00 h). The third flight trajectories were calculated by the same way as the second flight. Total fight duration of at maximum three flights was forced to be less than 36 h, in order not to overestimate the flight distance.

### Synoptic weather conditions for FAW migration

Since synoptic weather conditions have great impact on migration processes of high‐flying insects (Drake & Farrow, 1988; Westbrook & Isard, 1999; Hu *et al*., 2016, 2019), wind, temperature and sinking airflow during whole migration periods were analyzed. Based on meteorological data of NCEP‐NCAR, GrADS 2.1 software (http://cola.gmu.edu/grads/) was used to extract the average wind field, temperature, cumulative overnight rainfall (18:00–06:00 h) at 850 hPa (∼1500 m above sea level) between 2 June and 30 June 2019.

## Results

### Field surveys and estimated arrival dates for the Korean and Japanese cases

It has been reported for the first time that FAW's 1st–3rd instar larvae were found on Jeju Island, Korea, on 13 June 2019 (Fig. 1). Then, 3rd–5th instar larvae were found in Muan, Jeollanam Province, and Gochang, Jeonbuk Province, Korea, successively around 21 June, and 5th–6th instar larvae at many sites in southern coastal area Jeollanam, Jeonbuk and Gyeongsangnam Provinces around 28 June (Fig. 1, Table 1 and Table S1). Based on local ambient temperatures, probable arriving dates at each site were calculated (Table 1). The moths likely arrived on Jeju Island during 21–29 May and Gochang 31 May–7 June. Although these two sites are very close to each other, the probable arrival dates did not overlap (Table 1).

**Table 1:** Temporal relationships between field survey and trajectory analysis of *S. frugiperda* found in Korea and Japan in 2019

Locations	Date of caterpillars found	Instar	Direct degree‐day estimation^†^	Temporal range for trajectory analysis^‡^
Jeju Island	13 June	1–3	23–27 May	19–29 May^§^
Gochang, Jeonbuk	21 June	3–4	2–5 June	31 May–7 June
Minamikyushu, Kagoshima	27 June	5–6	6–11 June	27 May–13 June
Bungoohno, Ooita	12 July	5–6	20–25 June	7–27 June^¶^
Asagiri, Kumamoto	11 July	5–6	17–23 June	7–25 June
Miyakonojo, Miyazaki	12 July	5–6	16–21 June	6–23 June
Nakatane, Kagoshima	3 July	5–6	14–19 June	4–21 June
Onna, Okinawa	11 July	5–6	27–30 June	17 June–1 July

^†^ The latest day of the direct degree‐day estimation was calculated by assuming the oldest larva just molted from its previous instar, and the earliest day by assuming the oldest larva would molt into the next instar soon.

^‡^ The temporal range for trajectory analysis (i.e. probable arrival date) is the period of degree‐day estimation shifting 2 d earlier and 2 d later. In the Japanese cases, only old caterpillars were found in fields, but no young caterpillars. Therefore, it was suspected that some caterpillars already turned into the pupa stage and hid in the soil. As adults can survive at least approximately 10 d, the earliest degree‐day estimation was shifted by 10 d earlier as the first day for trajectory analysis.

^§^ No trajectories were found from Guangdong, Guangxi, Hainan and Yunnan Provinces during 22–29 May for this site, and trajectories were calculated for the other three previous days 19–21 May.

^¶^ No valid trajectories were found during 10–27 June for this site, and trajectories were calculated for during 7–9 June.

In Japan, 5th–6th instar larvae were for the first time found in Minamikyushu on 27 June 2019. In following days, 5th–6th instar larvae of the FAW were widely found during 3–11 July in Kyushu and on southwestern small islands such as Asagiri, Bungoohno, Miyakonojo, Nakatane and Onna (Fig. 1; Table S1).

Among these sites, FAW moths were suspected to arrive at Minamikyushu during 27 May–13 June (Table 1). For other sites in Kyushu and Nakatane on Tanegashima Islands, their probable arrival dates were all during around 7–20 June (Table 1). Because these sites were very close to each other, it seems that FAW arrived at these sites around 10 June as an immigration wave. Since, however, Onna in Okinawa Prefecture is located more south, where the air temperature was higher than other northern sites, the FAW moth was estimated to have arrived during 17 June–1 July, which was a little later than the other sites (Table 1). In summary, the immigrants in Japan were estimated to have come in two migration waves, northern and southern ones.

Taken the above results together, it was speculated that FAW moths probably migrated into Korea and Japan via three or four migration waves: (i) moths arrived on Jeju Island during 21–29 May; (ii) arrived at Gochang during 31 May–7 June; (iii) in Kyushu and on its surrounding islands around 10 June; and (iv) arrived on Okinawa Islands during 17–29 June.

As of 22 May 2019, FAW larvae were widely found in southern Anhui, Fujian, Zhejiang, Jiangxi and Hubei Provinces as well as in more southern provinces (red triangles in Fig. 1). This suggests that adult moths occurred in the region before 22 May. The larvae distribution expanded a little to the north by 5 June (blue solid triangles in Fig. 1), suggesting that adult moths already arrived in this region by early June. The region covers southern Anhui, southern Jiangsu and Zhejiang Provinces, and Shanghai as well as southern provinces. In Taiwan Province, the first larvae of 6th instar were found on cut feed corns in Miaoli County, western part of the island on 8 June 2019 (Bureau of Animal and Plant Health Inspection and Quarantine, 2019). By 20 June, the larvae boundary advanced further to the north between 33 and 35 °N (green solid triangles in Fig. 1), which are similar to latitudes of Korean and Japanese larvae sites (red and blue solid circles). The moths distributed in areas of less than 35 °N in three countries.

**Fig. 2:**
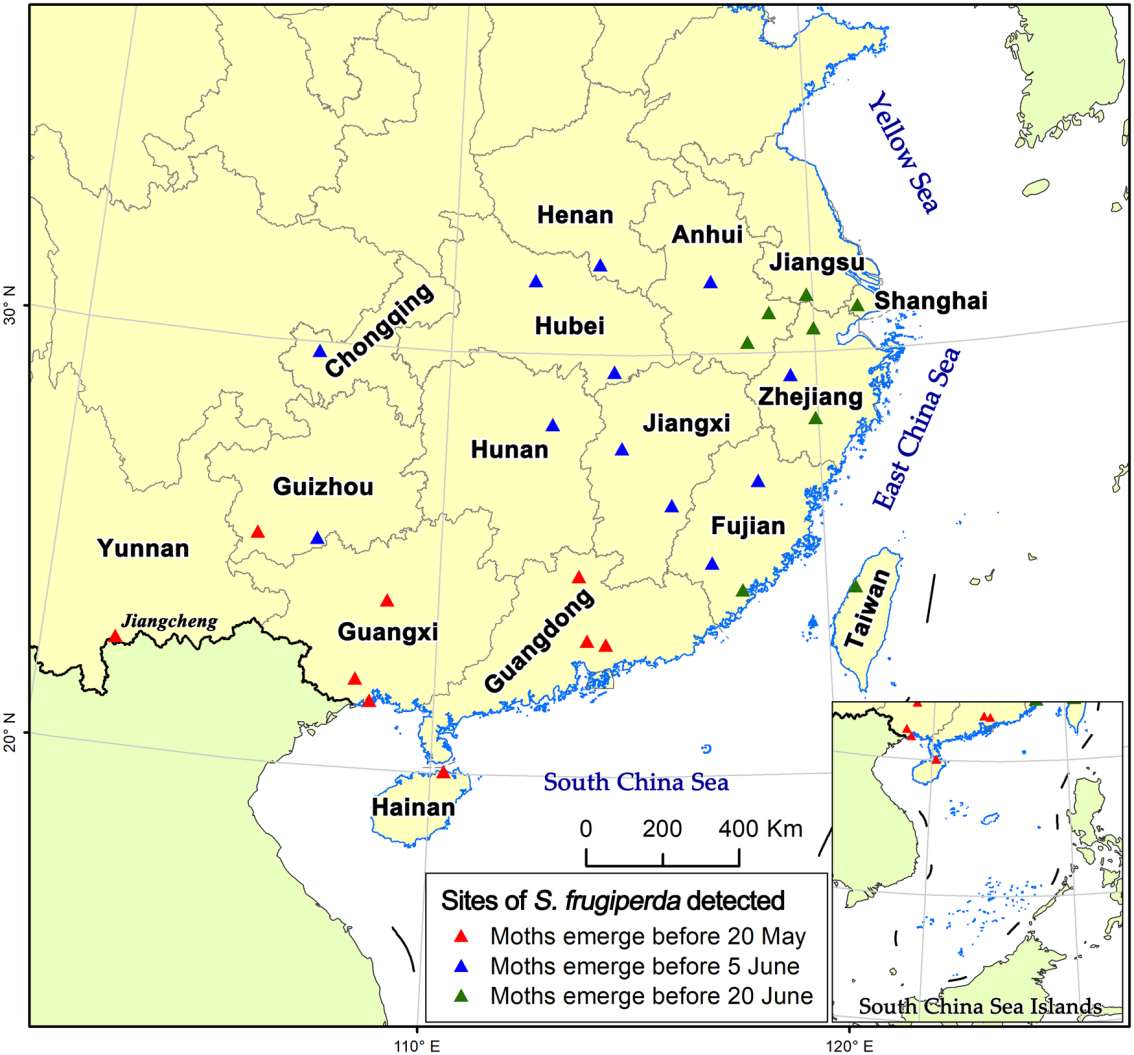
Possible emerging and emigrating dates of *S. frugiperda* estimated by the degree‐day model. GS(2021)3135.

The first caterpillars detected in each location in China are likely the offspring of immigrants from further south, and thus a site can be identified as an original source area only when new moths emerged from these caterpillars. Based on the degree‐day estimation, only in Yunnan, Guangxi, Guangdong and southwestern Guizhou Province FAW moths emerged and emigrated before 22 May (red triangles in Fig. 2). However, by 5 June, FAW moths would widely emerge in southern Anhui, Fujian, Zhejiang, Jiangxi and Hubei Provinces as well as in more southern provinces (blue triangles in Fig. 2). By 20 June, FAW moths would emerge in more northerly areas (green triangles in Fig. 2).

### Migration timing and possible source area estimated by backward trajectories

To find source areas for first and early immigrants in Korea and Japan, backward trajectories were calculated hourly on all of the probable arrival days for each site (Table 1). The exact arrival dates and times were estimated by the starting dates and times of valid backward trajectories, and these dates showed that FAW moths had migrated into Korea and Japan by three migration waves: 22–24 May, 5–10 June and 19–24 June (Table 2).

**Table 2:** Valid trajectories of *S. frugiperda* for the Korean and Japanese overseas migration cases

Locations	No. of valid trajectories	Estimated arrival date	Estimated arrival time(KST or JST: UTC + 9 h)	Simulated flight duration (h ± SD)^†^
Jeju Island	41	19 May	06:00, 07:00, 11:00–23:00	**24.8 ± 7.0**
	17	20 May	00:00–07:00	33.2 ± 2.1
	23	22 May	10:00–23:00	**20.7 ± 4.3**
	49	23 May	00:00–23:00	24.4 ± 7.7
	31	24 May	00:00–08:00, 13:00, 15:00–20:00	29.9 ± 5.6
Gochang	21	5 June	09:00–12:00	**14.4 ± 0.9**
	2	6 June	23:00	27.0
	6	7 June	00:00–02:00, 05:00–06:00	**19.3 ± 10.5**
Bungoohno	38	7 June	12:00–23:00	**20.2 ± 2.5**
Asagiri	52	7 June	12:00–23:00	**22.0 ± 3.6**
	3	8 June	00:00	28.0
Minamikyushu	121	7 June	12:00–23:00	**22.1 ± 4.4**
	50	8 June	00:00–8:00, 16:00–17:00, 21:00–22:00	29.9 ± 4.2
Miyakonojo	45	7 June	12:00–23:00	**23.0 ± 2.7**
	8	8 June	00:00–02:00	28.4 ± 0.8
	1	9 June	04:00	32.0
Nakatane	1	7 June	23:00	27.0
	18	8 June	00:00–08:00	32.1 ± 3.0
	6	10 June	00:00, 02:00–06:00	31.3 ± 2.2
Onna	28	19 June	14:00–23:00	**21.4 ± 2.6**
	2	20 June	23:00	27.0
	4	21 June	05:00–08:00	34.5 ± 1.4
	5	22 June	14:00–15:00	**18.2 ± 0.4**
	8	23 June	16:00–18:00, 21:00–23:00	23.6 ± 2.8
	9	24 June	01:00, 03:00, 08:00–10:00	24.9 ± 10.2

^†^ Bold typeface indicates the flight duration is less than 24 h, suggesting fast airstreams.

### First migration wave during 22–24 May: Jeju Island

**Fig. 3:**
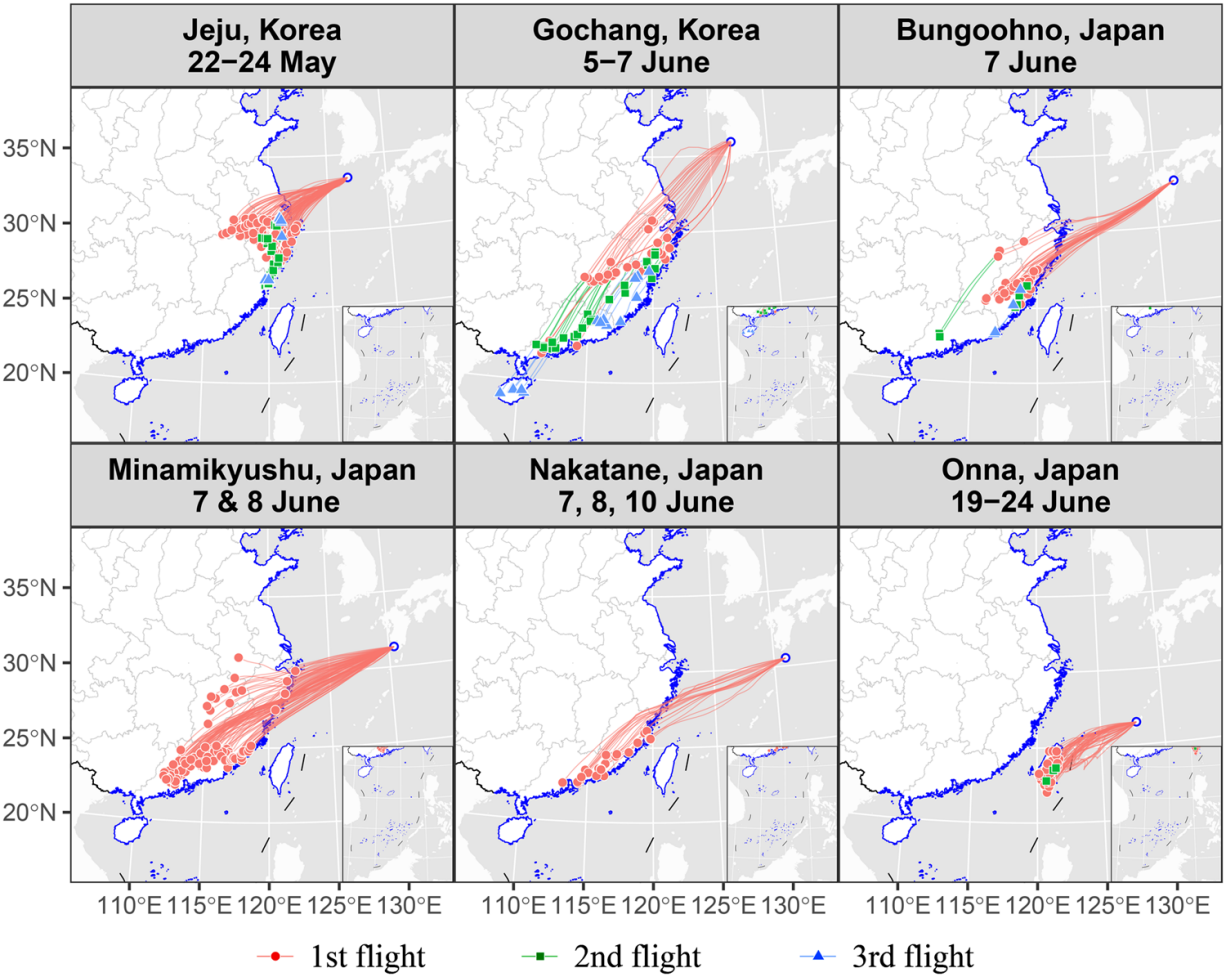
Simulated backward trajectories showed possible sources and migrating pathways for *S. frugiperda*’s larvae found in Korea and Japan in June and July 2019. Sites of caterpillars found (blue open circle: starting point of trajectories) and estimated arrival dates are labelled on the top of each panel. The trajectories of the first flight were calculated hourly on every probable arrival date from the degree‐day analysis, because exact times and dates when immigrants landed were unknown. Trajectories of the second and third flight starting from valid end‐points of the previous flight were then calculated as nocturnal flight over the Chinese mainland but each total flight duration was limited to be less than 36 h. This figure suggests that *S. frugiperda* moths entered Korea and Japan via three temporally different waves of overseas migration. GS(2021)3135.

Among backward trajectories from Jeju Island on the probable arrival date of FAW (i.e. 22–29 May), 103 valid trajectories were found on 22–24 May (Fig. 3; Table 2). These valid trajectories originated mostly from Zhejiang (75.7%) and South Anhui (19.4%) Provinces (Fig. 3; Table 3). The mean flight duration to cover these migration distances was 25.2 ± 0.7 h (range, 9–36 h). This result suggested that FAW moths from Zhejiang and Anhui took off between 21–23 May.

**Table 3:** Provinces where end‐points of valid trajectories for the 2019 Korean and Japanese overseas migration cases distributed

Locations	Date	Flight order	Provinces^†^	No. of valid end‐points (% in each flight)
Jeju	19–20 May	1st flight	Zhejiang	28 (48.3%)
			Fujian	27 (46.6%)
			Anhui	3 (5.2%)
		2nd flight	Zhejiang	16 (59.3%)
			Fujian	10 (37.0%)
			Jiangxi	1 (3.7%)
		3rd flight	Zhejiang	10 (41.7%)
			Fujian	7 (29.2%)
			Jiangxi	4 (16.7%)
			Guangdong	3 (12.5%)
Jeju	22–24 May	1st flight	Zhejiang	78 (75.7%)
			Anhui	20 (19.4%)
			Fujian	3 (2.9%)
			Jiangxi	2 (1.9%)
		2nd flight	Zhejiang	21 (61.8%)
			Fujian	13 (38.2%)
		3rd flight	Zhejiang	1 (100%)
Gochang	5‐7 June	1st flight	Jiangxi	13 (44.8%)
			Zhejiang	8 (27.6%)
			Guangdong	5 (17.2%)
			Fujian	3 (10.3%)
		2nd flight	Guangdong	13 (56.5%)
			Zhejiang	6 (26.1%)
			Fujian	4 (17.4%)
		3rd flight	Hainan	5 (35.7%)
			Guangdong	4 (28.6%)
			Fujian	4 (28.6%)
			Zhejiang	1 (7.1%)
Bungoohno	7 June	1st flight	Fujian	31 (81.6%)
			Jiangxi	4 (10.5%)
			Zhejiang	3 (7. 9%)
		2nd flight	Fujian	5 (71.4%)
			Guangdong	2 (28.6%)
Asagiri	7, 8 June	1st flight	Fujian	40 (72.7%)
			Jiangxi	10 (18.2%)
			Zhejiang	4 (7.3%)
			Guangdong	1 (1.8%)
		2nd flight	Fujian	4 (57.1%)
			Guangdong	3 (42.9%)
Minamikyushu	7, 8 June	1st flight	Guangdong	111 (64.9%)
			Fujian	36 (21.1%)
			Jiangxi	14 (8.2%)
			Zhejiang	8 (4.7%)
			Anhui	2 (1.2%)
Miyakonojo	7–9 June	1st flight	Fujian	40 (74.1%)
			Guangdong	8 (14.8%)
			Zhejiang	5 (9.3%)
			Jiangxi	1 (1.9%)
		2nd flight	Guangdong	1 (100%)
Nakatane	7, 8, 10 June	1st flight	Fujian	10 (40%)
			Guangdong	15 (60%)
Onna	19–24 June	1st flight	Taiwan	56 (100%)
		2nd flight	Taiwan	3 (100%)

^†^ Provinces where valid end‐points distributed.

After that, taking these valid end‐points as the departure points, backward trajectories were repeatedly calculated with other two continuous nights (for the previous two more nights), namely the 2nd and 3rd flight. There were 34 and one valid trajectory for the 2nd and 3rd flights, respectively. The end‐points of the 2nd flight were distributed mainly in Zhejiang (61.8%) and Fujian (38.2%) Provinces (Fig. 3; Table 3). The effective trajectory of 3rd flight was from Zhejiang Province (Fig. 3, Table 3).

All above valid end‐points were located in South Anhui, Zhejiang, Fujian and Jiangxi Provinces, where FAW caterpillars were detected by 22 May (Fig. 1). However, none of these caterpillars could molt into moths by 22 May (Fig. 2), and these places might not be the original source area, although adults had already arrived in the area at that time. Therefore, more trajectories were calculated for the other previous 3 d (19–21 May). Most of these trajectories (89.9% [89/109] of all valid end‐points) were from Zhejiang and Fujian (Table 3), but three of these effective trajectories of the 3rd flight were found from Guangdong Province (Table 3). This result suggested that FAW moths from Guangdong possibly migrated into Korea within 36 h, even if Zhejiang and Fujian were not directly original sources.

### Second migration wave during 5–10 June: southwestern Korea and western Japan

Valid backward trajectories from Gochang in Korea, and Bungoohno, Asagiri, Minamikyushu, Miyakonojo and Nakatane in Japan showed that estimated arrival dates and times at these sites were close to each other (Table 2), and this result suggested that moths arriving at these sites migrated from a same source area simultaneously and/or successively.

At Gochang, 29 valid backward trajectories were found on 5–7 June. During the 1st flight, end‐points of these trajectories were located in Jiangxi (44.8%), Zhejiang (27.6%), Guangdong (17.2%) and Fujian (10.3%) Provinces, and the mean simulated duration was 16.3 ± 1.1 h (range 9–30 h) (Fig. 3, Table 3). Among these valid trajectories, 23 trajectories were further extended to the 2nd flight, and 14 trajectories even to the 3rd flight. End‐points of the 2nd flight were located in Guangdong (56.5%), Zhejiang (26.1%) and Fujian (17.4%) Provinces, while end‐points of the 3rd flight were located far in Hainan (35.7%), Guangdong (28.6%) and Fujian (28.6%) Provinces.

In this migration wave, FAW moths landed on Kyushu Island and its neighboring Tanegashima Island widely. Backward trajectories were calculated for Bungoohno, Asagiri, Minamikyushu, Miyakonojo and Nakatane, and 343 valid trajectories appeared during 7–10 June. Valid end‐points of the 1st flight were located in Fujian (45.8%, 157/343), Guangdong (39.4%, 135/343), Jiangxi (8.5%, 29/343) and Zhejiang (5.8%, 20/343). The mean simulated duration of these trajectories was 24.0 ± 0.3 h (range, 16–36 h). Only 15 of these valid trajectories were further extended to the 2nd flight. Valid end‐points of the 2nd flight were located in Fujian (60%, 9/15) and Guangdong (40%, 6/15).

### Third migration wave during 19–24 June: Okinawa Island

Fifty‐six valid trajectories were appeared during 19–24 June, and all of the trajectories were from Taiwan Province. The mean simulated duration of these trajectories was 23.4 ± 0.7 h (range, 13–36 h). Only three of these valid trajectories were further extended to the 2nd flight, and all ended in Taiwan Province. Moreover, half of these 56 valid trajectories appeared on 19 June.

### Migrating heights

**Fig. 4:**
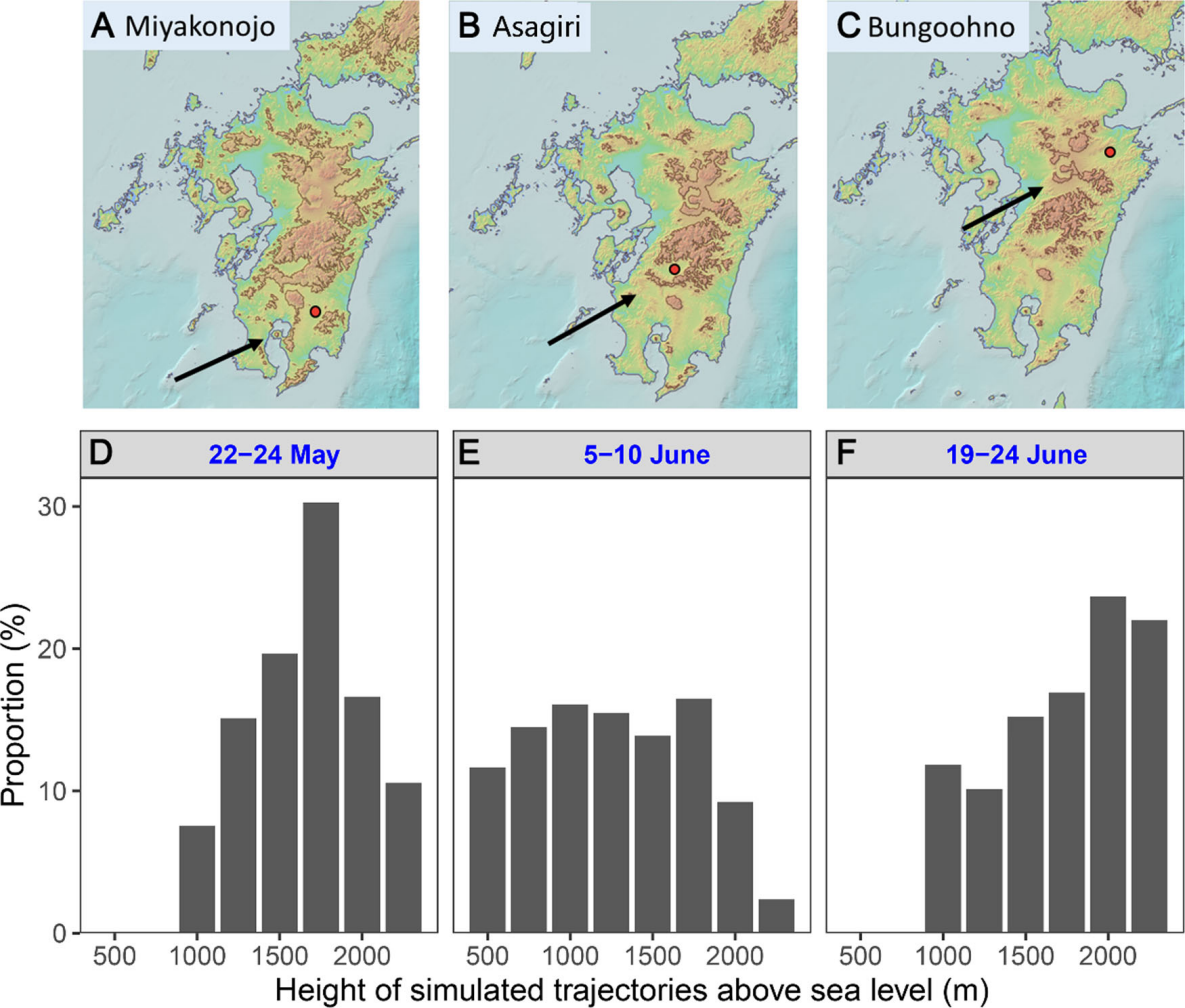
Topography of Kyushu Island, western Japan and site location (A–C), and histogram of height of simulated trajectories above sea level during three migration waves (D–F). The brown region indicated heights over 400 m (A), 650 m (B) and 800 m (C). A red circle shows locations where larvae were found. A black arrow indicates estimated immigration direction.

Valid overseas trajectories suggested that FAWs arrived in western Japan from the southwest direction (Fig. 3), which is a typical immigration direction in this region. There are mountains of various heights to the southwest of the sites, for example, Miyakonojo, Asagiri and Bungoohno (Fig. 4A–C), and FAW migrants should have flown over the top of topography (500–900 m). Specially, the height of mountain ridge to the southwest of Bungoohno is up to 900 m above sea level. These analyses suggested that the flight height of the immigrants ranged from near surface to over 900 m.

The trajectory analysis found that the height of valid backward trajectories in three migration waves ranged 500–2250 m (Fig. 4E–G) and that 73.8 % of the valid trajectories in the second wave (5–10 June) had flight heights of 1000 m or more, supporting a high‐latitude immigration over western Japan. Trajectories in the first migration wave during 22–24 May are concentered at the height of 1750 m, in the second wave 750–1750 m, and in the third wave 1500–2000 m.

### Synoptic weather during FAW migration period

**Fig. 5:**
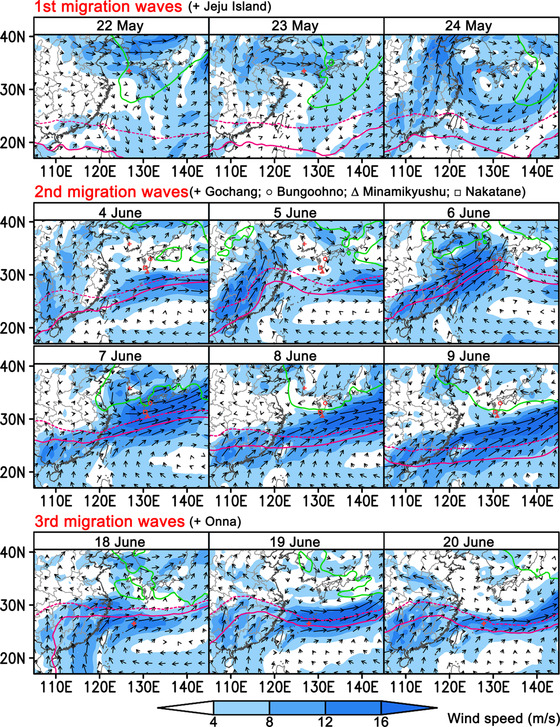
The wind pattern (vector shows wind direction and speed, and filled color shows wind speed) and temperature (green line shows 13.8 °C isotherm) at the level of 850 hPa (∼1500 m above sea level) around possible take‐off or arrival time during the migration period. The range of western Pacific subtropical high pressure is presented using the 500‐hPa geopotential height contour (geopotential decameter, gpdm). Red solid lines represent the 588‐gpdm contour, red dash lines represent the 586‐gpdm contour. GS(2021)3135.

Generally, a low‐level jet (LLJ, wind speed ≥12 m/s) is a fast‐moving narrow airstream in the low levels of the atmosphere (Hodges & Pu, 2019) and an ideal passageway for the long‐distance movement of migrating insects. Just as expected, strong eastward or northeastward LLJs were observed at 850 hPa (∼1500 m above sea level) to blow from eastern and southern China to Korea or Japan during all of the migration waves, and their wind speeds were even up to over 16 m/s (Fig. 5). This suggested that FAW migrated into Korea and Japan by riding on these high‐speed airstreams. It can be also found that the location of LLJs was related to the western Pacific high pressure (WPSH) during the second and third migration waves (Fig. 5). In general, LLJs were observed along the northwestern periphery of WPSH (Fig. 5) (Hu *et al*., 2019). Here we show a wind pattern in the second migration wave as an example. On 4 June, an LLJ began to form as WPSH began to strengthen and covered the area further north (Fig. 5). On June 5, the LLJ formed and covered southeastern China. Then, the strong wind area moved eastward in the next 2 d, reaching Korea and Japan. As the WPSH became weakened, the LLJ moved to the south a little and did not cover Korea and Japan anymore. During this period, FAW moths gradually reached the larval‐discovery sites from north to south. It was also found that the larval‐discovery sites were mostly located in the area of 13.8 °C or more (a green line in Fig. 5).

## Discussion

Fall armyworm caterpillars were reported to have been found in Korea and Japan in June 2019 for the first time. This study analyzed the migratory paths and weather backgrounds for the first and early immigration cases invading into Korea and Japan. The migration trajectory simulation implied that FAW immigrants producing larvae found in Korea in early June came mainly from Zhejiang, Jiangxi and Anhui Provinces when the single direct flight was assumed (Table 3). Similarly, immigrants responsible for the Japanese first and early cases in late June to early July were suggested to have come directly from Fujian, Jiangxi, Guangdong and Taiwan Provinces.

By 22 May, FAW caterpillars were already widely found in most parts of southeast China, such as southern Anhui, Zhejiang, Jiangxi and Fujian Provinces (Fig. 1). Additionally, according to field investigations conducted by plant protection and plant inspection stations in Jiangxi, Zhejiang, Fujian and Taiwan Provinces and the National Agro‐Tech Extension and Service Center of China, FAW occurred at varying degrees at Huangshan in Anhui Province (18 May), in Ganzhou in Jiangxi Province (7 May), Jiande in Zhejiang Province (8 May, Luo *et al*., 2020) and Quanzhou of Fujian Province (6 May) since early May 2019. This suggested that moths occurred in these areas before 22 May, indicating these areas could provide FAW immigrants into Korea and Japan. Moreover, based on the degree‐day estimation, the caterpillars that were first detected in the above area would molt into adults in early June (Fig. 2). Hence, most parts of southeast China could provide enough FAW immigrants into Korea and Japan after the development and reproduction of one generation in early June.

The discussion above indicated that most part of southern and eastern China including Anhui, Zhejiang, Fujian, Taiwan, Jiangxi, Guangdong and and Hainan Provinces could be possible sources of the FAW migrants to Korea and Japan at the time of estimated migrations in late May to early June. The inferred occurrence period of FAW moth was generally consistent with the estimated larval development duration, which indirectly confirmed the reliability of the trajectory analysis results in this study. These results were basically consistent with the results of the trajectory analysis.

For Japanese migration cases, no valid backward trajectories were found around the simple arrival dates directly estimated by the degree‐day method. Instead, valid trajectories were found approximately 10 d before those. This result suggests that the Japanese larvae were the very last offspring of the immigrants in the second migration wave. At the same time, it is also suggested that the immigrants could lay eggs even 10 d after the arrival.

This study for the first time estimated the flight height of FAW's overseas migrations, which ranged 500–2250 m. Previous radar studies of the migration of similar‐sized moths in China, indicating flight heights of nocturnal migrations over the flat plane, were around 200–300 m above the ground, which corresponds to the height of nocturnal winds of maximum speed (Feng *et al*., 2003, 2004, 2008). The estimated flight or arrival heights in this study are much higher than those over the land. When an overseas migration happens in the *Meiyu* rainy season (June to July) over the East China Sea, an LLJ blows in the south of a seasonal rain front, like the second migration wave case (Fig. 5). The height of the averaged jet core in early July 1969 over western Kyushu was located at around 700 hPa (= 3000 m) and its maximum horizontal wind speed was approximately 18 m/s (Matsumoto *et al*., 1971). Therefore, if a moth flies in the LLJ, the wind speed increases as the flight height increases (e.g. 8 m/s at 950 hPa [600 m], 12 m/s at 900 hPa [1000 m] and 14 m/s at 850 hPa [1500 m] in this 1969 case). Based on this structure of the LLJ stream, it is likely that the flight height of the FAW's overseas migrations over the East China Sea in 2019 was determined by the ambient air temperature of the flight, not by the maximum wind speed area like the cases over the land (Feng *et al*., 2003, 2004, 2008). This is one of the differences between the overseas and over‐the‐land migrations (the flight duration and distance of the overseas migrations are longer). This study used the temperature threshold of 13.8 °C as an assumed value. When this value is changed to higher, the flight height gets lower, greatly affecting trajectory results.

Based on the previous predictions (Ma *et al*., 2019), 11.6% of all the every‐night FAW trajectories during 1 June to 15 July in 2014–2018 with a dusk‐takeoff from Anhui and Zhejiang Provinces would invade South Korea, while 6.4% from Fujian and Zhejiang Provinces would invade Japan. This study's results were basically consistent with the previous predictions and actual occurrence trends of FAW. The inferred occurrence period of FAW moth was generally consistent with the estimated larval development duration, which indirectly confirmed the reliability of the trajectory analysis results in this study.

Due to the strong long‐distance migration ability of FAW, its surprisingly rapid spread and its significant capacity to generate high yield losses has generated worldwide concern. Within a year, FAW has spread through virtually all major corn‐producing regions of mainland China, Japan and Korean Peninsula. Several studies of seasonal migratory flight routes for FAW in Southeast Asia, China, Japan and Korean Peninsula are basically consistent with the current occurrence trends (Ma *et al*., 2019; Wu *et al*., 2019; Li *et al*., 2020). This study indicated that the FAW invade into Japan and Korean Peninsula in June or earlier; the results provide a basis and reference for predicting and controlling this pest, and we need to strengthen monitoring before this.

## Disclosure

All authors have seen and agree with the contents of the manuscript and there is no conflict of interest, including specific financial interest and relationships and affiliations relevant to the subject of the manuscript.

## Supporting information


**Table S1** Probable arrival date of *S. frugiperda* in all field survey sites in Korea and Japan.
