# Supplementary material for: Overseas immigration of fall armyworm, Spodoptera frugiperda (Lepidoptera: Noctuidae), invading Korea and Japan in 2019
Source: Insect Sci. 2021 Oct 15;29(2):505–20. doi: 10.1111/1744-7917.12940 (PMC9292357; doi:10.1111/1744-7917.12940)
Supplement: Supplementary file 1 — Table S1 Probable arrival date of S. frugiperda in all field survey sites in Korea and Japan. [file INS-29-505-s001.docx]

**Table S1** Probable arrival date of *S. frugiperda* in all field survey sites in Korea and Japan.

| Location^†^ | Latitude^‡^ | Longitude | Date of caterpillar found | Larva | Direct degree-day estimation | Probable arrival date^§^ |  |
| --- | --- | --- | --- | --- | --- | --- | --- |
| **Gochang, Jeonbuk** | **35.84** | **126.85** | **21 Jun** | **3–4** | **2–5 Jun** | **31 May–7 Jun** |  |
| Milyang, GN | 35.50 | 128.75 | 28 Jun | 5–6 | 30 May–4 Jun | 20 May–8 Jun |  |
| Muan, Jeollanam | 34.96 | 126.52 | 19 Jun | 4–5 | 24–30 May | 22 May–1 Jul |  |
| Boseong, Jeollanam | 34.77 | 127.08 | 28 Jun | 5–6 | 30 May–5 Jun | 20 May–7 Jun |  |
| Yeosu, Jeollanam | 34.75 | 127.66 | 28 Jun | 5–6 | 31 May–5 Jun | 21 May–7 Jun |  |
| Haenam, Jeonbuk | 34.57 | 126.60 | 28 Jun | 5–6 | 30 May–5 Jun | 20 May–7 Jun |  |
| **Gujwa, Jeju Island** | **33.49** | **126.75** | **13 Jun** | **1–3** | **23–27 May** | **21–29 May** |  |
| Sasebo, Nagasaki | 33.23 | 129.64 | 9 Jul | 5–6 | 19–23 Jun | 9–25 Jun |  |
| **Bungoohno, Ooita** | **32.99** | **131.60** | **12 Jul** | **5–6** | **20–25 Jun** | **10–27 Jun** |  |
| Koshi, Kumamoto | 32.88 | 130.75 | 8 Jul | 5–6 | 7–15 Jun | 28 May–17 Jun |  |
| Shimabara, Nagasaki | 32.82 | 130.34 | 11 Jul | 5–6 | 21–25 Jun | 11–27 Jun |  |
| Unzen, Nagasaki | 32.81 | 130.18 | 11 Jul | 5–6 | 21–25 Jun | 11–27 Jun |  |
| Yamato, Kumamoto | 32.67 | 130.99 | 11 Jul | 5–6 | 10–19 Jun | 31 May–21 Jun |  |
| **Asagiri, Kumamoto** | **32.23** | **130.91** | **12 Jul** | **5–6** | **17–23 Jun** | **7–25 Jun** |  |
| Kawaminami, Miyazaki | 32.21 | 131.52 | 16 Jul | 5–6 | 28–31 Jun | 18 Jun–2 Jul |  |
| Kishiro, Miyazaki | 32.16 | 131.46 | 12 Jul | 5–6 | 17–23 Jun | 7–25 Jun |  |
| Saito, Miyazaki | 32.09 | 131.39 | 10 Jul | 5–6 | 14–20 Jun | 4–22 Jun |  |
| Shintomi, Miyazaki | 32.08 | 131.47 | 13 Jul | 5–6 | 19–24 Jun | 9–26 Jun |  |
| Ebino, Miyazaki | 32.05 | 130.83 | 10 Jul | 5–6 | 14–20 Jun | 4–22 Jun |  |
| Kobayashi, Miyazaki | 32.02 | 130.98 | 10 Jul | 5–6 | 14–20 Jun | 4–22 Jun |  |
| Yushui, KG | 31.96 | 130.73 | 2 Jul | 5–6 | 4–10 Jun | 25 May–12 Jun |  |
| Satsuma, KG | 31.94 | 130.47 | 2 Jul | 5–6 | 10–15 Jun | 31 May–17 Jun |  |
| Takahara, Miyazaki | 31.93 | 131.00 | 8 Jul | 5–6 | 11–18 Jun | 1–20 Jun |  |
| Kirishima, KG | 31.80 | 130.77 | 1 Jul | 5–6 | 3–8 Jun | 24 May–10 Jun |  |
| **Miyakonojo, Miyazaki** | **31.76** | **131.07** | **11 Jul** | **5–6** | **16–21 Jun** | **6–23 Jun** |  |
| Soo, KG | 31.65 | 130.98 | 1 Jul | 5–6 | 3–8 Jun | 24 May–10 Jun |  |
| Hioki, KG | 31.55 | 130.34 | 29 Jun | 5–6 | 7–11 Jun | 28 May–13 Jun |  |
| Shibushi, KG | 31.52 | 131.09 | 1 Jul | 5–6 | 3–8 Jun | 24 May–10 Jun |  |
| **Minamisatsuma, KG** | **31.48** | **130.34** | **29 Jun** | **5–6** | **8–13 Jun** | **29 May–15 Jun** |  |
| Oosaki, KG | 31.44 | 130.99 | 1 Jul | 5–6 | 12–16 Jun | 2–18 Jun |  |
| Kanoya, KG | 31.43 | 130.87 | 1 Jul | 5–6 | 12–16 Jun | 2–18 Jun |  |
| Minamikyushu, KG | 31.32 | 130.44 | 27 Jun | 5–6 | 6–11 Jun | 27 May–13 Jun |  |
| Makurazaki, KG | 31.29 | 130.31 | 29 Jun | 5–6 | 8–13 Jun | 29 May–15 Jun |  |
| Ibusuki, KG | 31.20 | 130.62 | 1 Jul | 5–6 | 12–16 Jun | 2–18 Jun |  |
| Nishinoomote, KG | 30.72 | 131.03 | 3 Jul | 5–6 | 14–19 Jun | 4–21 Jun |  |
| **Nakatane, KG** | **30.53** | **130.96** | **3 Jul** | **5–6** | **14–19 Jun** | **4–21 Jun** |  |
| Kikai, KG | 28.33 | 129.97 | 5 Jul | 5–6 | 19–22 Jun | 9–24 Jun |  |
| Tokunoshima, KG | 27.83 | 128.95 | 4 Jul | 5–6 | 18–21 Jun | 8–23 Jun |  |
| Isen, KG | 27.72 | 128.92 | 4 Jul | 5–6 | 18–21 Jun | 8–23 Jun |  |
| Okinoerabu, KG | 27.37 | 128.57 | 8 Jul | 5–6 | 23–26 Jun | 13–28 Jun |  |
| **Onna, Okinawa** | **26.50** | **127.85** | **11 Jul** | **5–6** | **27–30 Jun** | **17 Jun–1 Jul** |  |

^†^KG-Kagoshima; GN-Gyeongsangnam.

^‡^Latitude and longitude of Japanese locations stand for those of an arbitrary point in the city, town, or village where larvae were found. They don’t indicate the exact location of the collection points.

^§^The temporal range of probable arrival date is the period of degree-day estimation shifting 2 days earlier and 2 days later. In the Japanese cases, only old caterpillars were found in fields, but no young caterpillars. Therefore, it was suspected that some caterpillars already turned into pupa stage and hid in soil. As adults can survive at least about 10 days, the earliest degree-day estimation was shifted by 10 days earlier as the first day for probable arrival date. Grey cell color indicates the sites had a detail analysis in main text.
